# Precision Medicine and Triple-Negative Breast Cancer: Current Landscape and Future Directions

**DOI:** 10.3390/cancers13153739

**Published:** 2021-07-26

**Authors:** Fokhrul Hossain, Samarpan Majumder, Justin David, Lucio Miele

**Affiliations:** 1 Department of Genetics, Louisiana State University Health Sciences Center (LSUHSC), New Orleans, LA 70112, USA; smaju1@lsuhsc.edu (S.M.); lmiele@lsuhsc.edu (L.M.); 2 Stanley S. Scott Cancer Center, Louisiana State University Health Sciences Center (LSUHSC), New Orleans, LA 70112, USA; 3 School of Medicine, Louisiana State University Health Sciences Center (LSUHSC), New Orleans, LA 70112, USA; jdav45@lsuhsc.edu

**Keywords:** triple-negative breast cancer (TNBC), precision medicine, breast cancer, targeted therapy, TNBC subtypes, immunotherapy

## Abstract

**Simple Summary:**

The implementation of precision medicine will revolutionize cancer treatment paradigms. Notably, this goal is not far from reality: genetically similar cancers can be treated similarly. The heterogeneous nature of triple-negative breast cancer (TNBC) made it a suitable candidate to practice precision medicine. Using TNBC molecular subtyping and genomic profiling, a precision medicine-based clinical trial is ongoing. This review summarizes the current landscape and future directions of precision medicine and TNBC.

**Abstract:**

Triple-negative breast cancer (TNBC) is an aggressive and heterogeneous subtype of breast cancer associated with a high recurrence and metastasis rate that affects African-American women disproportionately. The recent approval of targeted therapies for small subgroups of TNBC patients by the US ‘Food and Drug Administration’ is a promising development. The advancement of next-generation sequencing, particularly somatic exome panels, has raised hopes for more individualized treatment plans. However, the use of precision medicine for TNBC is a work in progress. This review will discuss the potential benefits and challenges of precision medicine for TNBC. A recent clinical trial designed to target TNBC patients based on their subtype-specific classification shows promise. Yet, tumor heterogeneity and sub-clonal evolution in primary and metastatic TNBC remain a challenge for oncologists to design adaptive precision medicine-based treatment plans.

## 1. Precision Medicine: Perspective and Challenges

The human genome project opened a path to understanding human gene structure and function and identifying disease-associated mutations in our DNA. Since the human genome project, there have been dramatic advancements in genetic technology, with continuous progress towards more cost-efficient and powerful techniques [1]. The development of the chip-based microarray allowed early gene expression profiling studies as well as genome-wide association studies for millions of single nucleotide polymorphisms (SNPs). Still, it was eventually replaced for most non-SNP applications by the high-throughput next-generation sequencing (NGS) of genomic DNA and RNA-derived cDNA [2]. These advancements have paved the way for genomic medicine. Genomic medicine is an integral part of precision medicine, defined by the NIH as an emerging approach of tailoring treatment and prevention based on individual variability in genes, environments, and lifestyle to classify individuals into specific subgroups susceptible to one particular treatment plan [3]. Although some think of precision medicine and personalized medicine interchangeably, the two concepts are not identical. “Precision” medicine uses data and genomics to tailor treatments to specific groups sharing genetic and/or clinical, environmental and lifestyle features. “Personalized” medicine would imply treatments designed specifically for individual patients. Except in unique circumstances, such as tumor vaccines or tumor-infiltrating lymphocytes (TIL) produced from individual tumors, truly personalized medicine remains an aspirational goal. In 2015, US President Barack Obama announced an NIH-funded precision medicine initiative to assemble the most significant medical research cohort in history, collecting health and behavioral data as well as DNA and other biospecimens from one million or more Americans reflecting the diversity of our population. The centerpiece of the precision medicine initiative is the “All of US” research program [3]. This program is expected to collect and share data from genome sequencing, electronic medical records, personal reported information, and digital health technologies [3]. Using these integrated datasets, researchers will assess the effectiveness of treatments and identify genetic variations associated with a higher or lower risk of disease or adverse medication events of any particular group of people. This information can form the basis for the design of novel biomarker studies and therapeutic trials. In effect, the goal is to stimulate a progressive transition away from generalized, broad-spectrum therapies to more precise treatments in well-defined patient populations [4].

Knowing the genetics of diseases will allow physicians to make health care decisions that are more effective for the patient to improve the quality of care and decrease unnecessary screenings or procedures [3]. For example, genetic analysis revealed that there are subgroups of type I and II diabetes that differ in medication responsiveness due to genetic differences. Thiazolidinedione use has declined, but genetic analysis could identify patients who are more likely to respond to this group of drugs [5]. Genome-wide association studies (GWAS) analyze human DNA variation to identify risk factors and improve treatment strategies [2]. One of the first GWAS studies identified polymorphisms in the cytochrome P450 2C9 (CYP2C9) complex and the vitamin K epoxide reductase complex 1 (VKORC1), which are both correlated with Warfarin pharmacokinetics [6]. Pharmacogenomics studies have revealed several other genetic variants associated with differential drug metabolism [7,8,9]. Despite advancements in precision medicine, there are many obstacles to its routine clinical deployment. One significant barrier to the clinical use of genomic data is the high number of variants of unknown significance in the human genome and the difficulty in attaining sufficiently large sample sizes to analyze their possible roles [10]. Additionally, identifying risk factors is not always straightforward, as the relationship between risk factors and their biology is often more complex than previously anticipated. Two patients with the same risk factors do not necessarily share the same disease [11]. There is also difficulty in identifying the standard features of polymorphisms between ancestral groups. For instance, African genomes are more polymorphic and have less linkage disequilibrium in single nucleotide polymorphisms than Europeans [2]. Another barrier to the advancement of precision medicine is the lack of infrastructure and education in clinical-based settings. Routine precision medicine practice will require highly integrated patient datasets, including clinical, lifestyle, and genetic data [3], and there is a lack of genetics professionals in hospitals [12]. Finally, cost and reimbursement issues must be solved before fully integrating genetic data into the clinical setting [12,13]. Solutions to these barriers include developing technology infrastructure, outcome-based reimbursement policies, education and promotion to personnel in clinical-based settings [14], and patient willingness to participate in precision medicine [3]. There is a promising future for precision medicine, but it must overcome a number of obstacles before it is fully integrated into the healthcare system.

One field that is currently benefiting from the development of clinical genomics is oncology. Many new cancer therapies [4] use precision medicine and genomic tests based on NGS as a strategy to identify cancers that are more likely to respond, as opposed to anatomical sites. Whole-exome sequencing (WES) helped discover and understand driver mutations and copy number variants in cancers, and WGS is slowly improving our limited knowledge of mutations in non-coding regions [15]. One of the earliest therapeutics developed based on genetic alterations was trastuzumab for cancers carrying genomic amplification of a region of chromosome 17 containing the ERBB2/HER2 gene, which led to better outcomes than first-line chemotherapy [16]. Subsequently, genetic analysis revealed that cetuximab effectively treated colorectal cancers in patients without KRAS mutations [17]. Targetable prostate cancer mutations have been lacking, but PARP-1 inhibition has been effective in certain patients [18]. The discovery of specific mutations can advance the development of therapeutics for the treatment of other cancers with the same mutation, though context can make a difference. For example, trastuzumab also benefits gastric cancers with HER2 amplification [19]. In contrast, BRAF inhibitors were effective in hairy cell leukemia with BRAF mutations but not in colorectal cancer, highlighting the limitations of single gene-based approaches and the importance of clinical trials for targeted therapy [20]. Prevention strategies have also been proposed for patients with a genetic predisposition to some cancers, such as sulindac and celecoxib causing polyp regression in patients with Familial Adenomatous Polyposis [21]. Despite the development of potentially effective therapies based on mutational profiles, tumor heterogeneity adds another barrier to precision medicine in oncology. The clonal heterogeneity of tumors and the evolution of clones carrying additional mutations compared to the original drivers is a significant obstacle to the effectiveness of targeted cancer therapies, particularly as monotherapy. Frequently, after targeted treatment based on a driver mutation produced clinical responses, the tumor will circumvent the targeted pathway blockade through genetic evolution or epigenetic plasticity and will find a way to resume progression [22]. Clonal heterogeneity is often based on mutational heterogeneity, with some cells showing specific mutations, while other cancer cells display different mutational profiles [23]. Evolutionary “trees” of tumor clones under the form of “tropical fish plots” effectively show this phenomenon [24]. Usually, the development of new mutations under therapy-imposed selection results in treatment resistance [25]. One group of cancers that would greatly benefit from precision oncology is triple-negative breast cancer (TNBC).

## 2. Triple-Negative Breast Cancer (TNBC)

Triple-negative breast cancer (TNBC) is an aggressive and heterogeneous subtype of breast cancer. TNBC was found to be negative for the estrogen receptor α (ER−), the progesterone receptor (PR−), and the human epidermal growth factor receptor two loci (HER2−) by immunohistological analysis [26,27,28,29,30]. TNBC constitutes 11–20% of all breast cancers and typically affects premenopausal women, especially African American women [26,30,31]. TNBC has a higher rate of mortality and recurrence than other types of breast cancer, especially in the first five years [26]. There are currently a few targeted therapies available for TNBC, but chemotherapy remains the mainstay of treatment, while immunotherapy is a recent and increasingly important addition [27,31,32,33]. Optimizing the treatment of TNBC based on genomic and possibly immunological features would be a significant advancement.

Comorbidities have significant effects on the risk and outcomes of TNBC, in part by affecting tumor biology. Obesity is linked with increased incidence and a worse prognosis of triple-negative breast cancer [34]. One theory of obesity’s relation to TNBC biology is that obesity increases the development of a pro-inflammatory and metabolically activated phenotype of macrophages (MMe). MMe macrophages are dominant in obese human and mouse mammary adipose tissue. They are tumorigenic due to the increased secretion of IL-6 in a NADPH oxidase-2 (NOX2)-dependent fashion. IL-6 signals through glycoprotein-130 induce stem-like properties in TNBC cells [34]. Another study concluded that the increased inflammation and reactive oxygen species from obesity drive the increased expression of a splicing variant of methyl-CpG-binding domain 2 (MBD2_v2), increasing the stem cell-like properties of TNBC cells [35]. Increased adipose tissue caused by obesity also increases the secretion of the hormone leptin, which enhances the expression of genes linked to stem cell-like properties and epithelial–mesenchymal transition [36]. Another theory is that hyperinsulinemia secondary to insulin resistance increases the activation of the AKT/mTOR pathway, promoting proliferation and survival in TNBC cells. Additionally, the AKT/mTOR pathway increases glucose uptake and promotes the Warburg effect, a shift from aerobic oxidation in the mitochondria to anaerobic glycolysis, which allows for rapid growth and resistance to apoptosis [37,38,39]. The development of targeted therapies for TNBC must consider these factors, including the cross-talk between the adipose tissue, metabolism, and tumor biology.

The progression of a primary tumor to a metastatic tumor is based on its ability to leave the original site and spread into the blood and/or the lymphatic system, potentially forming new tumors in other locations in the body. Breast tumors start in the mammary ducts or lobules but can spread into the surrounding adipose tissue and migrate to other parts of the body, escaping immune surveillance mechanisms [40]. Metastatic TNBC is more aggressive compared to other breast cancers, and the average rate of patient survival is lower than other subtypes [41]. Understanding the biological differences between a primary TNBC tumor and a metastatic TNBC tumor could benefit therapy. Primary TNBC is associated with relatively few somatic single-nucleotide variants, but numerous somatic copy number variations (CNV) [42]. The cell cycle’s loss of function mutations and the apoptosis regulator p53 [42,43,44,45] as well as the gain-of-function PIK3CA mutations are common in primary TNBC [43,44]. However, the possible mutational landscape for TNBC is very broad and contains numerous other genes associated with the control of cell shape, motility, and extracellular signaling [43]. Metastatic TNBC is associated with p53, LRP1B, HERC1, CDH5, RB1, and NF1 mutations in general [45]. The biology of the progression from a primary tumor to metastasis is not completely understood. Comprehensive gene expression profiles in primary and metastatic breast cancer revealed many differentially expressed genes in metastatic versus primary disease [46]. Metastatic breast cancer, including metastatic TNBC (mTNBC), is a major concern in the inpatient treatment regimen. Recent advancements in immunotherapy and targeted therapies in breast cancer improve the longevity of cancer patients. In addition to the advancement of targeted therapies, locoregional resection also plays an important role in preventing metastasis [47,48]. Further understanding of the genetic landscape associated with primary and secondary TNBC is an urgent need.

## 3. TNBC Subtypes and Current Treatment Options

**Figure 1:**
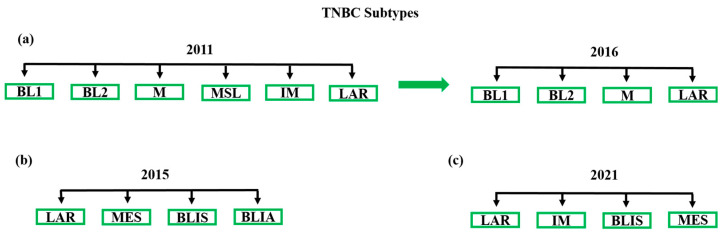
Major TNBC subtypes based on gene expression profiles. (**a**) Lehmann et al. classified TNBC patients into six subtypes (TNBCtype) in 2011: basal-like 1 (BL1), basal-like 2 (BL2), mesenchymal (M), mesenchymal stem-like (MSL), immunomodulatory (IM), and luminal androgen receptor (LAR) [52]. In 2016, Lehman et al. re-classified the TNBC molecular subtypes from six (TNBCtype) to four (TNBCtype-4) tumor-specific subtypes: BL1, BL2, M, and LAR [56]. (**b**) Burstein el al. suggested four subtypes: luminal androgen receptor (LAR), mesenchymal (MES), basal-like immunosuppressed (BLIS), and basal-like immune-activated (BLIA) [54]. (**c**) FUTURE trial schema: LAR, immunomodulatory (IM), mesenchymal-like (MES), and basal-like immune-suppressed (BLIS) [57].

TNBC molecular subtypes are a useful starting point on the road to TNBC precision treatment [26,27,28,29,30,31,49,50,51]. Based on the gene expression profiles of TNBC samples, Lehmann et al. classified TNBC patients into six subtypes: basal-like 1 (BL1), basal-like 2 (BL2), mesenchymal (M), mesenchymal stem-like (MSL), immunomodulatory (IM), and luminal androgen receptor (LAR) [52]. These authors also developed a web-based subtyping tool (TNBCtype) to predict subtype assignment for new TNBC samples to guide biomarker or treatment studies [53]. Subsequently, Burstein et al. classified TNBC into four subtypes: LAR, M, BLIS (basal-like immunosuppressed), and BLIA (basal-like immune-activated) [54]. They reported that the prognosis was the worst for BLIS tumors and the best for BLIA tumors in terms of disease-free survival (DFS) and disease-specific survival (DSS). The order from best to worst prognosis was BLIA > M > LAR > BLIS for both DFS and DSS [54]. Liu et al. classified TNBC tumors based on the expression profiles of both mRNAs and lncRNAs and proposed the Fudan University Shanghai Cancer Center (FUSCC) classification as well as the analysis of its interaction with the Lehman/Pietenpol subtypes [55]. They divided TNBC tumors into four subtypes including the immunomodulatory subtype (IM), the mesenchymal-like subtype (MES), the luminal androgen receptor subtype (LAR), and the basal-like and immune-suppressed (BLIS) subtype [55]. In 2016, Lehmann et al. re-classified the TNBC molecular subtypes from six (TNBCtype: (BL1, BL2, IM, M, MSL and LAR)) to four (TNBCtype-4) tumor-specific subtypes (BL1, BL2, M, and LAR) based upon the complexity and overlapping of the varying histological landscapes of tumor samples [56]. The IM and MSL subtypes were dependent upon transcripts from immune infiltrates and other tumor stromal cells. While these transcripts may affect tumor biology, their reproducibility was linked to levels of tumor infiltration. These authors demonstrated that TNBC subtypes had a significantly different response to neo-adjuvant chemotherapy and suggested that the classification will benefit future clinical trial design [56]. Different TNBC subtype classifications are presented in Figure 1.

TNBC has higher rates of early recurrence and mortality than other types of breast cancer. The reason for the poor outcomes of TNBC is the lack of effective targeted therapies. Endocrine agents such as aromatase inhibitors or HER2 targeted monoclonal antibodies or small molecules are not effective for TNBC patients [58,59,60,61]. Therefore, standard cytotoxic chemotherapy (doxorubicin, docetaxel, 5-fluorouracil, platinum drugs, and/or cyclophosphamide and other agents in different combinations) remains the standard of care for TNBC patients [62,63,64,65]. It is essential to consider the risks and benefits of treating early-stage TNBC patients. Over-treatment increases toxicity and undesirable adverse effects, compromising the patient’s quality of life. Early-stage TNBC patients without lymph node involvement generally have a good prognosis in terms of five-year relapse-free survival (RFS) and five-year distant recurrence-free survival (DRFS) [66,67]. Chemotherapy is the choice of care for TNBC patients with a tumor size > 5 mm with or without lymph node (LN) metastases. Combinations of anthracyclines, alkylators, and taxanes with carboplatin are common chemotherapy regimens for TNBC [68]. The ABC trial suggested that the addition of an anthracycline to docetaxel and cyclophosphamide therapy significantly improves invasive disease-free survival (IDFS) for early stage TNBC patients [69].

In the adjuvant (post-surgical) setting, the treatment of eight weeks of paclitaxel followed by the standard regimen of adjuvant fluorouracil, epirubicin, and cyclophosphamide (FEC) decreased tumor relapse and improved DFS in LN-positive breast cancer [70]. Neoadjuvant chemotherapy (NACT) is now used as the standard of care to treat high-risk TNBC to reduce tumor volume before surgery [71,72,73,74,75,76,77,78,79]. Patients treated with standard NACT have approximately 30–40% pathologic complete response rates (pCR) [49,80,81,82]. Tumors that do not achieve pCR have significantly higher recurrence rates than tumors that do. The ability to predict which patients achieve pCR and/or to increase pCR rates without increasing toxicity would be major advances in the treatment of TNBC. Tumor-infiltrating lymphocytes (TIL) within residual tumors post-NACT are considered as a semi-quantitative assessment of immune response [83,84,85,86,87,88,89,90]. In a landmark study, Denkert et al. [91], analyzed the results of the GEPAR-Sixto clinical trial and, determined that increased levels of stromal TILs predicted pCR. Gene expression profiling revealed three immune subtypes with different pCR rates. The most predictive transcripts were PD-L1 and CCL5. Subsequent studies confirmed the predictive value of immunophenotyping and TILs, suggesting a role of the immune system in clearing tumor cells during chemotherapy [92]. Another gene signature consisting of HLF, CXCL13, SULT1E1, and GBP1 in pre-treatment samples predicted the extent of lymphocytic infiltration after NACT [93]. Tumor mutational burden, possibly resulting in higher numbers of tumor-associated antigens, was identified as an independent predictor of pCR in addition to TIL [94]. Conversely, the presence of a PIK3CA H1047R mutation was associated with lower rates of pCR [95].

Following NACT, the most commonly used prognostic factor in TNBC is pCR. However, pCR is not an absolute predictor, as some TNBC patients who achieve pCR develop relapses [96,97,98,99]. Studies from the MD Anderson Cancer Center have reported a more quantitative evaluation scale called the Residual Cancer Burden (RCB), which is based on tumor size, invasive cancer cellularity, and node status post-NACT [100,101]. RCB is classified on a 0-III range, with the higher values indicating the probability of subsequent recurrence, metastatic spread, and increased mortality.

## 4. Recently FDA-Approved Therapies for TNBC

Chemotherapy and surgery remain the standard of care for most TNBC patients. However, a few classes of agents, such as Immune Checkpoint Blockers (ICBs), PARP inhibitors (PARPi), and Antibody Drug Conjugates (ADC), have demonstrated clear benefits in TNBC patients, and in some cases, have received FDA approval. Nonetheless, response rates with the new agents are variable, and predicting the response to these new classes of agents will be the focus of a major precision oncology effort.

### 4.1. Approved Checkpoint Inhibitors

Many TNBC tumors are immunologically “cold”, meaning they lack sizeable TIL infiltrates (see above for biomarkers of tumor immunity). Converting “cold” TNBC to “hot” tumors and making them amenable to treatment with ICBs would be a potentially valuable treatment strategy. Approximately 40% of TNBC expresses PD-L1 in TILs, and PD-L1 positive tumors (PD-L1 positivity is defined by PD-L1 expression on tumor-infiltrating immune cells covering ≥1% of the tumor area) tend to respond favorably to treatment with anti-PD-L1 therapy [102,103,104]. In an analysis of a PD-L1-positive TNBC cohort, the addition of atezolizumab, a humanized monoclonal antibody to PD-L1, in combination with nab-paclitaxel compared to chemotherapy (nab-paclitaxel)-alone significantly improved median progression-free survival (PFS) to 7.5 months versus 5.0 months, respectively (HR 0.62; 95% CI, 0.49–0.78), and improved OS to 25 months from 15.5 months, respectively (HR, 0.62; 95% CI, 0.45–0.86) [105]. Based on this compelling data, on 8 March 2019, the FDA granted the accelerated approval of atezolizumab (Tecentriq) in combination with nab-paclitaxel (Abraxane) for the treatment of PD-L1-positive unresectable locally advanced and metastatic TNBC tumors [106]. The FDA also granted accelerated approval to Merck’s anti-PD-1 monoclonal antibody, pembrolizumab (Keytruda), in combination with chemotherapy for locally recurrent or metastatic TNBC in November, 2020 [107]. A first-line treatment regimen of pembrolizumab with chemotherapy extended PFS by 35% compared to a placebo. However, the FDA declined to grant accelerated approval to Keytruda in either neoadjuvant or adjuvant settings for high-risk, early-stage TNBC. The FDA panel reasoned that a 15% increase in pCR would not necessarily be indicative of an increase in overall survival (OS).

#### Other Immune Checkpoint Inhibitors in Clinical Trials

A recent paper described details about clinical trials involving PD-1/PD-L1 blockade either as monotherapy, in combination with chemotherapy, or with other targeted therapies [108]. Gagliato et al. also described the success and challenges of PD-1/PD-L1 immunotherapy for TNBC patients [109]. In addition to PD-1 and its ligand (PD-L1), other immune checkpoint inhibitors are also being investigated in TNBC clinical trials, including cytotoxic T lymphocyte-associated protein 4 (CTLA-4), Lymphocyte-activation gene 3 (LAG-3), and T cell immunoglobulin and mucin-domain containing-3 (TIM-3) [110]. Ipilimumab, a CTLA4 blocking antibody, is in a phase 2 TNBC clinical trial with nivolumab, a PD-1 blocking antibody with taxane-based neoadjuvant chemotherapy (Clinicaltrials.gov: NCT03546686). Another phase 2 clinical trial is ongoing with nivolumab in combination with ipilimumab for advanced or metastatic solid tumors, including TNBC (Clinicaltrials.gov: NCT01928394). Tremelimumab, another anti-CTLA-4 monoclonal antibody, is in a phase I clinical trial with durvalumab (anti-PD-L1 monoclonal antibody) in combination with chemotherapy in advanced solid tumors, including TNBC (Clinicaltrials.gov: NCT02658214). Tremelimumab is also being tested in phase 2 clinical trials as a monotherapy or with MEDI4736 for advanced solid tumors, including in TNBC (Clinicaltrials.gov: NCT02527434).

Bottai et al. suggested that LAG-3 and PD1 were co-expressed in approximately 15% of TNBC patients, and their co-expression positively correlated with the presence of tumor-infiltrating lymphocytes (TILs) [111]. LAG525 (IMP701), an anti-LAG-3 antibody in combination with spartalizumab (an anti-PD-1 checkpoint inhibitor) is under a phase I clinical investigation in patients with advanced or metastatic TNBC (Clinicaltrials.gov: NCT03742349). TSR-033, an anti-LAG-3 monoclonal antibody, is in a phase 1 clinical trial alone and in combination with the anti-PD-1 antibody dostarlimab in patients with advanced solid tumors (Clinicaltrials.gov: NCT03250832). Another anti-LAG-3 antibody (INCAGN02385) is under a phase I clinical investigation in patients with advanced malignancies, including TNBC (Clinicaltrials.gov: NCT03538028). TIM-3, another immune checkpoint, plays an important role in tumor immunity [112]. An anti-TIM-3 antibody, INCAGN02390, is in phase I clinical trials in select advanced malignancies, including TNBC (Clinicaltrials.gov: NCT03652077).

### 4.2. Poly-ADP-Ribose Polymerase (PARP) Inhibitors

The loss of function mutations in the BRCA1 and BRCA2 genes have long been known to confer a high risk of TNBC. The loss of BRCA1/BRCA2 function mutations impairs DNA double-stranded break (DSB) repair in normal cells, leading to the accumulation of genetic damage and chromosomal aberrations. About 19.5% of TNBC cases are associated with germline BRCA1/BRCA2 gene mutations [113]. Cancer cells that are defective in DSB repair are susceptible to other mechanisms of DNA damage. Poly-ADP-Ribose Polymerase (PARP) is an enzyme involved in single strand break (SSB) DNA repair. Cells with DSB repair defects are vulnerable to SSBs, which trigger apoptosis. PARP inhibitors (PARPi) exploit this vulnerability. PARPi are well-tolerated and improve both progression free survival (PFS) and OS in TNBC patients with germline BRCA1/BRCA2 mutations, which is reviewed in [114,115]. The FDA approved two PARP inhibitors to treat TNBC patients with BRCA-mutant tumors. In January 2018, olaparib (Lynparza) was approved for the treatment of patients with BRCA-positive, HER2-negative metastatic breast cancer [116]. Talazoparib (Talzenna) was also approved in the same year for the treatment of patients with BRCA-mutated and HER2-negative locally advanced or metastatic breast cancer [117].

### 4.3. Antibody-Drug Conjugates (ADC)

Antibody-Drug Conjugates (ADC) are chemically modified monoclonal antibodies (mAb) usually linked to high-potency cytotoxic payloads. In such cases, the mAb is used to selectively target the toxic payload of cancer cells. ADCs are one of the fastest-growing classes of cancer therapeutics in the past few decades [118]. In 2020, the FDA granted accelerated approval to sacituzumab govitecan, an ADC sold under the brand name Trodelvy [119]. Accelerated approval was granted for the treatment of adults with TNBC that has metastasized and has received at least two prior therapies. Sacituzumab govitecan (Trodelvy) received regular approval from the FDA on 7 April 2021 for unresectable locally advanced or metastatic TNBC (mTNBC) [120]. Trodelvy is a Trop-2-directed antibody conjugated to govitecan, a topoisomerase inhibitor. However, despite mAb-mediated selective delivery, this agent has significant toxicity that has prompted a boxed warning: severe neutropenia and severe diarrhea are common. Patients experiencing neutropenia are advised to receive treatment with G-CSF to stimulate bone marrow hematopoiesis. A recent review paper highlighted the use of the ADCs, including sacituzumab govitecan (SG), ladiratuzumab vedotin (LV), and trastuzumab deruxtecan (T-DXd) in mTNBC [121]. Although T-Dxd has proven efficacy in HER2-overexpressing metastatic breast cancer, it has also shown better clinical responses in patients with low HER2-expressing metastatic breast cancers, including TNBC [121]. A phase Ib clinical trial (Clinicaltrials.gov: NCT04556773) is currently recruiting patients with metastatic HER2-low advanced or metastatic breast cancer to evaluate the efficacy of T-DXd in combination with other therapies.

## 5. Precision Medicine in TNBC: Emerging Therapies and Ongoing Studies

### 5.1. Receptor Tyrosine Kinases (RTKs) and Downstream Signaling Pathways

RTKs in TNBC signaling operates through two main downstream signaling cascades: the RAS/MAPK and the PI3K/AKT/mTOR signaling axis. RTKs in TNBC cells transduce signals downstream of EGFR, PDEGFR, VEGFR, IGFR, TGF-β, and FGFR. Almost 60–80% of TNBC tumors have dysregulated EGFR expression [122]. EGFR expression is associated with aggressive TNBC. Post NACT, EGFR expression frequently persisted in TNBC, suggesting that anti-EGFR therapy may offer an additional window of opportunity for patients with therapy-refractory EGFR-positive TNBC tumors [123]. The KRAS/SIAH/EGFR pathway is frequently upregulated in TNBC. The seven in absentia homolog (SIAH), an E3 ligase and the most downstream “gatekeeper” of the EGFR/KRAS signaling cascade, is often upregulated in TNBC along with EGFR [58,123]. Paired gene expression of SIAH and EGFR has been proposed as a prognostic biomarker in TNBC [123]. A decrease in SIAH and EGFR expression in a patient-derived specimen post-NACT compared to pre-NACT levels predicts treatment benefits.

Targeting EGFR would thus appear to be a potentially attractive strategy for TNBC. However, EGFR inhibitors have significant off-target toxicities [124], and multi-center clinical trials have not shown cetuximab, an anti-EGFR, to be an effective therapy for TNBC, probably due to the activation of compensatory signaling mechanisms such as PI3K-AKT (see below) [58].

Lapatinib, a dual EGFR/HER2 RTK inhibitor effective in HER2- positive breast cancer, was not effective in TNBC [125]. The MEK inhibitor selumetinib blocked the motility and invasiveness of the MDA-MB-231 and SUM149 TNBC cell lines in vitro [122]. Furthermore, selumetinib appeared to decrease lung metastasis in a TNBC-bearing mouse xenograft model [126], supporting the study of MEK inhibitors in TNBC. Compared to monotherapy, combining MEK inhibition with PD-L1/PD-1 inhibition increased therapeutic efficacy in a murine syngeneic TNBC model [127].

### 5.2. PI3K/AKT/mTOR Targeted Therapy

The PI3K/AKT/mTOR pathway is one of the most active cell survival pathways in cancer, often leading to chemoresistance [128]. This pathway, which is initiated by PI3K family kinases and receives input from EGFR family receptors, insulin, and insulin-like growth factor receptors, is a significant player in regulating apoptosis and metabolism. It also perpetuates the effect of BRCA mutations by stabilizing DNA double-stranded breaks [68]. PI3K/AKT/mTOR pathway dysregulation frequently occurs in TNBC. The PI3KCA-gain of function mutations is observed in 23.7% of TNBC patients [129]. Notably, the loss of function mutations or epigenetic silencing of the gene encoding PI3K negative regulator phosphatase PTEN, including promoter silencing and functional suppression, are detected in 25–30% of TNBC cases [122]. AKT and mTOR hyperactivation portend a poor prognosis in TNBC patients. The dual inhibition of AKT and mTOR (necessary to avoid feedback activation of AKT by mTORC2 after inhibition of mTORC1) may offer a promising therapeutic option for TNBC treatment [122,130] based on preclinical results. Investigational AKT inhibitors like ipatasertib and capivasertib have demonstrated incremental benefits in improving outcomes for patients with high-risk TNBC [131,132]. The FDA-approved mTORC1 inhibitor everolimus increases progression-free survival when combined with the non-steroidal aromatase inhibitor exemestane for patients with HR(+) and HER2(−) advanced breast cancer [133]. Everolimus combined with carboplatin has been proposed as an effective therapy for metastatic TNBC patients [134,135] because of the link between mTOR activation and platinum resistance [136]. PI3K inhibition can also decrease the expression of BRCA1 and 2 and can sensitize BRCA1/2 wild-type TNBC tumors for PARP inhibition [137]. Based on this observation, a clinical trial with BKM120 (buparlisib) and olaparib was initiated [122]. Interestingly, resistance to mTOR inhibitors in TNBC was accompanied by the appearance of Notch-dependent cancer stem-like cells (CSCs) [138], suggesting that Notch inhibitors may be combined with mTOR/AKT inhibitors.

### 5.3. Notch Signaling and TNBC

Notch signaling activation is correlated with TNBC tumor growth, CSCs maintenance, expansion, tumor invasiveness, and metastasis [139,140,141,142]. Notably, ~10% of TNBC carry driver Notch1/Notch2 chromosomal rearrangements that produce constitutively active, oncogenic forms of Notch1 or Notch2 [143]. Efforts to target Notch signaling in cancers, including in TNBC, have been ongoing for the past 15 years. However, an FDA-approved Notch inhibitor/drug is still elusive, though at least one of them is currently in phase 3. For an extensive review of the field, readers are directed to [142]. An abundance of preclinical data provided a compelling case for the use of gamma-secretase inhibitors (GSIs) to inhibit Notch signaling and reverse tumor progression in TNBC. However, GSI demonstrated mechanism-based dose-dependent gastrointestinal (GI) toxicities that limited their clinical applications [142,143]. Intermittent dosing of GSIs to circumvent their GI toxicities has been adopted in numerous clinical trials. Still, we do not know whether intermittent dosing is sufficient to eliminate TNBC CSCs [142,143]. Non-GSI, new generation Notch inhibitors are in clinical development. Among them, CB-103 appears particularly promising.

CB-103 (Cellestia biotech, Basel, Switzerland; https://www.cellestia.com/, accessed on 16 July 2021) is a first-in class, oral pan-Notch small molecule inhibitor with a unique mode of action. CB-103 inhibits the Notch transcriptional complex assembly and blocks Notch signaling in receiving cells [142,143]. Importantly, CB-103 does not induce mechanism-based dose-dependent GI toxicity, unlike GSIs. Furthermore, its action mechanism implies that CB-103 would be effective against the truncated forms of Notch1 or Notch2 produced by genetic rearrangements associated with ~10% of TNBC. The safety and efficacy of CB-103 in Notch-dependent advanced or metastatic solid tumors or hematological malignancies are being investigated in a phase I/II clinical trial (Clinicaltrials.gov: NCT03422679).

### 5.4. Cyclin-Dependent Kinases (CDKs)

Cyclin-dependent kinases (CDKs) play an essential role in modulating cell division by regulating cell cycle and transcriptional activities. Similar to many tumors, the aberrant expression of CDKs (e.g., CDK4 and CDK6) are also common in TNBC. CDK inhibitors have been used successfully to inhibit TNBC growth in preclinical settings. Dinaciclib, a pan-CDK inhibitor, is in phase I clinical trial in combination with pembrolizumab (Clinicaltrials.gov: NCT01676753) for TNBC and advanced or metastatic breast cancer. Trilaciclib, a CDK 4/6 Inhibitor, is in phase 1 clinical trial in combination with gemcitabine and carboplatin for metastatic TNBC (mTNBC) (Clinicaltrials.gov: NCT02978716). Ribociclib, another CDK 4/6 Inhibitor, is in phase I/II clinical trial in combination with bicalutamide, an androgen receptor (AR) inhibitor for advanced AR+ TNBC (Clinicaltrials.gov: NCT03090165). A phase II study of PF-06873600 (CDK inhibitor) in combination with endocrine therapy is ongoing for metastatic breast cancer, ovarian cancer, and TNBC (NCT03519178). Another phase II study of abemaciclib (selective ATP-competitive inhibitor of CDK4 and CDK6 kinase activity) for TNBC is also ongoing (Clinicaltrials.gov: NCT03130439). CDK inhibitors (mainly CDK4/6) augment anti-tumor immunity through T-cell activation, supporting the rationale for combination with immunotherapy [108,144,145]. A phase Ib clinical breast cancer study of pembrolizumab with abemaciclib demonstrated tolerability with clinical benefits [108].

### 5.5. Androgen Receptor (AR) Expression and TNBC

There is a lack of consensus among breast cancer researchers on whether AR expression is a favorable prognostic indicator in TNBC [146]. AR expression is upregulated in 10–43% of the TNBCs andfalls into the LAR molecular subtype. Therapy with AR antagonists showed clinical benefits in some TNBC patients [58,147,148,149,150]. A phase II randomized clinical trial is currently investigating the efficacy of a new AR antagonist, darolutamide, for unresectable or metastatic TNBC patients (Clinicaltrials.gov: NCT03383679) [58].

### 5.6. Angiogenesis and TNBC

Angiogenesis is one of the necessary adaptations that cancer cells must adopt to form macroscopic tumors. VEGF-A is the most critical pro-angiogenic secretory factor produced by solid tumors (see [58,151] for recent reviews). Elevated VEGF expression in TNGC is linked to poor prognosis independent of tumor size, histological grade, or nodal status [152]. The use of an anti-VEGF antibody, bevacizumab, in combination with chemotherapy was shown to improve PFS but failed to provide statistically meaningful improvements in OS in TNBC compared to chemotherapy alone [58,153]. The combined inhibition of VEGF and Notch ligand DLL4, which is also required for angiogenesis, through bispecific monoclonal antibodies is currently being investigated [142].

### 5.7. Investigational Antibody-Drug Conjugates (ADC)

Following the FDA approval of Trodelvy in 2020, several pharmaceutical companies are interested in ADC for TNBC treatment. In addition, the phase I study demonstrated favorable efficacy and tolerability using ladiratuzumab vedotin [118]. Recent review papers described other ADCs under clinical investigations [118,154].

## 6. Other Targeted Therapies

Recent progress in preclinical studies with small-molecule agents for the targeted therapy of TNBC has been summarized in recent publications [28,110,155]. Islam et al. described targeting the Bcl-2 family, proteasome, STAT3, histone deacetylase (HDACs), Src in TNBC in detail [155]. P53 mutations are prevalent in TNBC, which lead the cells to rely on checkpoint kinase 1 (ChK1) and ataxia telangiectasia related to Rad3 (ATR) for DNA repair management [110]. LY2606368, a ChK1/2 inhibitor, is under a phase 2 clinical trial for various tumors (ovarian, breast, and prostate), including TNBC (Clinicaltrials.gov: NCT02203513). Another phase 2 clinical trial is ongoing with olaparib in combination with an ATR inhibitor (ceralasertib) and adavosertib for metastatic TNBC patients (Clinicaltrials.gov: NCT03330847).

### 6.1. Cancer Vaccines

Apart from ICBs, an immunotherapeutic strategy that has been in development for decades but has yet to achieve its full clinical potential is cancer vaccines. Despite preclinical successes and the safety and immunogenicity in humans that have been documented in many clinical trials [156], cancer vaccines have yet to produce meaningful and reproducible clinical responses in TNBC [157]. This may be due to immune editing, a phenotypic and genetic adaptation process whereby cancers evade the immune system [157] and/or insufficiently robust T cell responses [156]. Perhaps the rapid evolution in vaccine technology sparked by the COVID19 pandemic, which borrowed from the field of cancer immunology, will eventually produce clinically effective cancer vaccines. Table 1 lists the active clinical trials from Clinicaltrials.gov as of 18 June 2021.

### 6.2. Combination Regimens

The molecular heterogeneity and frequent multi-clonality of TNBC strongly suggest that precision combination therapies are more likely to be successful than monotherapeutic strategies. The use of targeted agents plus standard chemotherapy or other targeted agents has shown promising results. One study found that olaparib, a PARP inhibitor, combined with the PI3K inhibitor buparlisib and carboplatin caused cytotoxic effects by promoting of non-homologous DNA end joining in TNBC cells [158]. A phase I study is assessing an olaparib–buparlisib combination regimen in TNBC and ovarian cancer (Clinicaltrials.gov: NCT01623349). PARP inhibitors have also shown activity with ADCs and chemotherapy, such as olaparib with sacitizumab govetican in TNBC cells with and without BRCA mutations [159] and iniparib in combination with gemcitabine and carboplatin [160]. PARPi have also been explored in combination with immunotherapy. PARPi have been shown to upregulate PD-L1, and PD-L1 inhibitors were documented to restore breast cancer sensitivity to PARP inhibitors [156]. In contrast, Higuchi et al. found no improvement of the efficacy of PARPi in BRCA-negative ovarian cancer with PD-1/PD-L1 inhibition, but they [161] did find improvement if PARPi were used in combination with CTLA-4 inhibitors [161]. Buparlisib in combination with DSF/Cu (Disulfiram/copper) and paclitaxel caused decreased tumor burden and recurrence rates in TNBC compared to paclitaxel alone [162]. In addition, a clinical trial involving the AKT inhibitor ipatasertib combined with paclitaxel has shown promising results (Clinicaltrials.gov: NCT02162719).

Combination therapy involving Notch targeting may be an attractive strategy. A late clinical development stage GSI, PF-03084014 combined with the AKT inhibitor MK-2206 or the NF-kB inhibitor Bay11-7082 effectively treated TNBC cells with a Notch mutation and wild-type PTEN [163]. Another study found a correlation between Notch3 inhibition and the increased effectiveness of the tyrosine kinase inhibitor gefitinib targeting EGFR in TNBC cells [164].

Combinations of immunotherapeutics with chemotherapy or with targeted agents are potentially promising. The I-SPY trial found success in combining the PD-1 inhibitor pembrolizumab with paclitaxel (Clinicaltrials.gov: NCT01042379), and the KEYNOTE 173 trial is seeing antitumor activity with pembrolizumab and neoadjuvant chemotherapy (Clinicaltrials.gov: NCT02622074). Another clinical trial is investigating PD-1 (nivolumab) and CTLA-4 inhibitors (ipilimumab) together along with cryoablation (Clinicaltrials.gov: NCT02833233). Many potential immunogenic tumors do not respond to immune checkpoint blockers. Kim et al. found significant improvement in outcomes when ICBs were used in combination with epigenetic-modulating drugs targeting myeloid-derived suppressor cells (MDSCs) or a PI3K inhibitor that reduced MDSCs [165].

## 7. Design of Precision Medicine-Based Clinical Trials in TNBC: The Path Forward

The future of TNBC developmental therapeutics depends on increasingly precise, biomarker-based, adaptive clinical trials. A perfect example is the ongoing FUTURE trial (Clinicaltrials.gov: NCT03805399) [57]. This is a phase I/II subtyping-based and genomic biomarker-guided umbrella trial where the investigators have classified metastatic TNBC patients based on molecular subtyping and genomic profiling. Patient classification for this trial relies on an integrative analysis that combines somatic mutations, copy number aberrations (CNAs), and gene expression profiles as well as validated immunohistochemistry surrogates. Based on these criteria, TNBC patients are stratified into four subtypes: (1) luminal androgen receptor (LAR), (2) immunomodulatory (IM), (3) basal-like immune-suppressed (BLIS), and (4) mesenchymal-like (MES) [57]. Within the LAR group, patients with HER2 mutations are treated with pyrotinib and capecitabine, while patients without HER2 mutations are treated with an androgen receptor antagonist and a CDK4/6 inhibitor. For the IM group, patients are treated with anti-PD-1 immunotherapy plus nab-paclitaxel. If the patients have BRCA1/2 germline mutation within the BLIS group, they are treated with a PARP inhibitor. If no BRCA1/2 germline mutations are detected, the patients are treated with anti-VEGFR therapy. Within the MES group, if patients have PI3K/AKT pathway mutations, they are treated with an mTOR inhibitor (Everolimus) with nab-paclitaxel. In contrast, patients without PI3K/AKT pathway mutations are treated with anti-VEGFR therapy. New arms can be added or existing arms can be terminated based on the ongoing examination of the study results. This type of study design is likely to become standard in TNBC and beyond.

## 8. Conclusions

The rapid evolution of targeted therapy and immunotherapy guided by somatic and in some cases germline genomics gives us realistic hopes for the more effective, more precise, and less toxic treatment of TNBC in the near future. However, further research is necessary to realize the full potential of precision medicine in TNBC. Somatic NGS alone has limitations, and we must move beyond simplistic one-gene-one therapy paradigms. Mutational landscapes change with time, and a single NGS test performed before treatment may not be representative of the tumor after chemotherapy or radiation. Longitudinal testing, if accessible tumor or perhaps circulating tumor cells are available, is likely to provide more accurate information. Many genotyped tumors reveal no targetable mutations, or, even when such mutations are identified, the patient may not respond to treatment [166]. This may be due to compensatory mutations, epigenetic changes, phenotypic plasticity, or clonal heterogeneity. Despite these limitations, genomic-driven therapy is already improving outcomes. An MD Anderson study found a higher response rate and more prolonged survival when treating single-mutation cancers with matched therapy [167].

Phenotypic screening using 3D tumor organotypic spheroids [168] may offer a promising alternative or complementary strategy, provided that results can be obtained rapidly, and treatment can potentially be adapted to tumor evolution using more than one round of screening. Pauli et al. were able to improve drug sensitivity screening using 3-D cultures and PDX models, showing that integration of exome sequencing with these methods could help better identify the best therapy [166]. An important limitation of these methods is that they require accessible tumor tissue. “Liquid biopsy,” a group of evolving methods to obtain circulating tumor cells or circulating tumor DNA from patient blood, could be an avenue for therapeutic screening and the longitudinal monitoring of molecular tumor profiles [169]. Single-cell RNA sequencing to identify resistant clones is now a reality [170]. Proteogenomics combining proteomic and genomic results is an attractive strategy if costs can be brought down [171]. The study of tumor metabolism and metabolomics is another highly promising precision medicine field that can be combined with genomics. A recent manuscript described a targetable retinoblastoma tumor suppressor, (RB1)-glucose transporter 1 (GLUT1) metabolic axis, in TNBC and suggested targeting GLUT1 in TNBC patients based on their RB1 protein expression levels [172]. The promise of precision medicine in the treatment of TNBC and other solid tumors is undeniable, and combinations of increasingly sensitive “omics” and phenotypic screening may significantly accelerate the development of novel therapeutics.

## Figures and Tables

**Table 1:** Active TNBC clinical trials using targeted therapy and Immunotherapy (Clinicaltrials.gov, accessed on 18 June 2021).

Title	Phase	Status	Age	ID
Evaluation of IPI-549 combined with front-line treatments in patients with TNBCr or Renal Cell Carcinoma (MARIO-3)	Phase II	Active	18 and over	NCT03961698
Testing the addition of Copanlisib to Eribulin for the Treatment of Advanced-Stage Triple Negative Breast Cancer	Phase I/II	Active	18 and over	NCT04345913
Study of Pembrolizumab (MK-3475) plus chemotherapy vs. placebo plus chemotherapy for previously untreated locally recurrent inoperable or metastatic TNBC	Phase III	Active, not recruiting	18 and over	NCT02819518
Atorvastatin in treating patients with stage IIb-III TNBC who did not achieve a PCR after receiving neoadjuvant chemotherapy	Phase II	Active	18 and over	NCT03872388
A Study of Atezolizumab in combination with Nab-Paclitaxel compared with placebo with Nab-paclitaxel for participants with previously untreated metastatic TNBC	Phase III	Active, not recruiting	18 and over	NCT02425891
Avelumab With Binimetinib, Sacituzumab Govitecan, or Liposomal Doxorubicin in treating patients with stage IV or unresectable recurrent TNBC (InCITe)	Phase II	Active	18 and over	NCT03971409
A Study of Atezolizumab and Paclitaxel versus placebo and paclitaxel in participants with previously untreated locally advanced or metastatic TNBC (Impassion131)	Phase III	Active, not recruiting	18 and over	NCT03125902
A Study evaluating the efficacy and safety of multiple immunotherapy-based treatment combinations in patients with metastatic or inoperable locally advanced TNBC (Morpheus-TNBC)	Phase I/II	Active	18 and over	NCT03424005
A Study of Cobimetinib plus paclitaxel, Cobimetinib plus Atezolizumab plus Paclitaxel, or Cobimetinib plus Atezolizumab plus Nab-Paclitaxel as initial treatment for participants with TNBC that has spread	Phase II	Active, not recruiting	18 and over	NCT02322814
Women’s MoonShot: Neoadjuvant treatment with PaCT for patients with locally advanced TNBC	Phase II	Active	18 and over	NCT02593175
A phase II study of Nivolumab in combination with Cabozantinib for metastatic TNBC	Phase II	Active, not recruiting	18 and over	NCT03316586
FUSCC Refractory TNBC Umbrella (FUTURE)	Phase I/II	Active	18 to 75 years	NCT03805399
Peri-Operative Ipilimumab + Nivolumab and Cryoablation in women with TNBC	Phase II	Active	18 and over	NCT03546686
Trilaciclib (G1T28), a CDK 4/6 Inhibitor, in Combination with Gemcitabine and Carboplatin in metastatic TNBC	Phase II	Active, not recruiting	18 and over	NCT02978716
